# Antibiotic Alternatives and Next-Generation Therapeutics for *Salmonella* Control: A One Health Approach to Combating Antimicrobial Resistance

**DOI:** 10.3390/antibiotics14101054

**Published:** 2025-10-21

**Authors:** Mohamed Saleh, Ashutosh Verma, Khaled A. Shaaban, Yosra A. Helmy

**Affiliations:** 1 Department of Veterinary Science, Martin-Gatton College of Agriculture, Food, and Environment, University of Kentucky, Lexington, KY 40546, USA; 2 Richard A. Gillespie College of Veterinary Medicine, Lincoln Memorial University, Harrogate, TN 37752, USA; 3 Department of Pharmaceutical Sciences, College of Pharmacy, University of Kentucky, Lexington, KY 40536, USA; 4 Center for Pharmaceutical Research and Innovation, College of Pharmacy, University of Kentucky, Lexington, KY 40536, USA

**Keywords:** *Salmonella*, antimicrobial resistance, next-generation therapeutics, One Health

## Abstract

The growing prevalence of antimicrobial resistance has significantly compromised the efficacy of conventional antibiotic-based interventions in controlling *Salmonella* infections across human and veterinary settings. This growing challenge necessitates a strategic rethinking of pathogen control, prompting the integration of next-generation therapeutics capable of disrupting *Salmonella* pathogenesis through novel, antibiotic-sparing mechanisms. In this context, a diverse array of emerging alternatives, including bacteriophages, antimicrobial peptides, probiotics, prebiotics, short-chain fatty acids, nanoparticles, and host-directed immunomodulators, have gained prominence as a promising frontier in non-antibiotic therapeutics. These modalities offer targeted approaches to inhibit *Salmonella* colonization, virulence expression, and persistence, while minimizing collateral damage to the microbiota and avoiding the propagation of resistance genes. As *Salmonella* continues to pose a global threat to animal and public health, the development of scalable, resistance-conscious interventions remains a critical priority. Ongoing research efforts are increasingly focused on optimizing delivery systems, dosage strategies, and synergistic combinations to enhance the clinical and field applicability of these alternatives. By harnessing these innovative modalities, the future of *Salmonella* control may shift toward precision therapeutics that align with One Health principles and sustainable food safety goals.

## 1. Introduction

Salmonellosis is a globally significant zoonotic disease with substantial implications for public health, food safety, and veterinary medicine [1,2]. It is caused by *Salmonella* spp., a genus of Gram-negative, facultatively anaerobic, rod-shaped bacteria belonging to the *Enterobacteriaceae* family [3,4]. These pathogens are taxonomically diverse, encompassing over 2600 serotypes, and are broadly categorized into typhoidal and non-typhoidal serovars based on their host specificity and clinical manifestations [5]. Typhoidal strains, such as *Salmonella* Typhi and *Salmonella* Paratyphi, are human-restricted and responsible for invasive systemic infections, particularly in regions with inadequate sanitation infrastructure [6]. In contrast, non-typhoidal *Salmonella* (NTS) serovars include *Salmonella* Enteritidis, *Salmonella* Typhimurium, and *Salmonella* Heidelberg exhibits a broad host range and is predominantly associated with self-limiting gastroenteritis in humans, as well as subclinical or clinical infections in livestock and poultry [2,7,8].

NTS serovars are frequently transmitted through contaminated food products, especially those of animal origin, and are a leading cause of bacterial foodborne illness worldwide. Their persistence in agricultural environments, ability to colonize asymptomatic carriers, and resilience against standard sanitation practices pose ongoing challenges to containment [2,9]. In humans, salmonellosis typically presents as acute gastroenteritis characterized by diarrhea, fever, abdominal cramps, nausea, and vomiting, with symptoms appearing 6–72 h post-exposure and lasting 4–7 days [10]. While most cases are self-limiting, vulnerable populations such as infants, the elderly, and immunocompromised individuals may develop invasive infections, including bacteremia, meningitis, osteomyelitis, and septic arthritis [10,11]. *Salmonella* infections can affect a wide range of animals, including poultry (chickens, turkeys, ducks), cattle, pigs, sheep, goats, horses, reptiles (such as turtles and lizards), amphibians, rodents, and companion animals like dogs and cats [2]. In animals, clinical signs vary by species and age, with young poultry and livestock exhibiting anorexia, diarrhea, and systemic signs such as hepatosplenomegaly [2,12].

Numerous foodborne outbreaks have prompted large-scale recalls of contaminated products, including eggs, cucumbers, deli meats, and chocolate spreads [13,14,15,16]. Recent recalls linked to *S. enteritidis* and *S. newport* have affected multiple states and food categories, underscoring the need for robust surveillance and traceability systems [17,18].

The therapeutic landscape has grown increasingly complex due to the global rise of multidrug-resistant (MDR) *Salmonella* strains. Resistance to traditional antibiotics such as ampicillin, chloramphenicol, tetracyclines, and trimethoprim-sulfamethoxazole has been widely documented, particularly in serovars associated with livestock and poultry production [8,9]. These MDR strains often harbor mobile genetic elements, including plasmids, integrons, and transposons, which facilitate horizontal gene transfer and accelerate the dissemination of resistance determinants across bacterial populations [19,20]. Alarmingly, resistance to extended-spectrum β-lactams and fluoroquinolones has also emerged in animals and humans, narrowing therapeutic options and prompting the need for enhanced surveillance, stewardship, and alternative treatment strategies [21,22,23].

This review aims to critically evaluate emerging non-antibiotic strategies for the control of multidrug-resistant *Salmonella*, with a particular focus on interventions such as probiotics, prebiotics, organic acids, bacteriophages, essential oils, antimicrobial peptides, small molecules, quorum sensing inhibitors, and vaccines. By synthesizing current evidence on their mechanisms of action, efficacy, and applicability across human and veterinary settings, this article discusses their potential to reduce pathogen colonization, enhance host immune responses, and curb the spread of antimicrobial resistance. The overarching goal is to inform integrated, sustainable approaches to *Salmonella* management that align with One Health principles and address the limitations of conventional antibiotic therapies.

## 2. Antibiotic Alternatives

### 2.1. Small Molecules (SMs)

Small molecules are low molecular weight organic compounds that can easily diffuse across cell membranes and modulate biological processes [22,24,25]. They may be naturally occurring (like microbial metabolites or plant alkaloids) or synthetically produced, and are often used as pharmaceutical drugs, research tools and/or signaling agents [23,26,27]. SMs have demonstrated the ability to inhibit bacterial growth by targeting key molecular pathways and disrupting intracellular processes [28,29]. In the context of *Salmonella* infections, they offer a promising therapeutic strategy by attenuating bacterial growth and virulence while minimizing the emergence of antibiotic resistance. These compounds can interfere with quorum sensing, biofilm formation, and essential metabolic functions [2,22].

**Figure 1:**
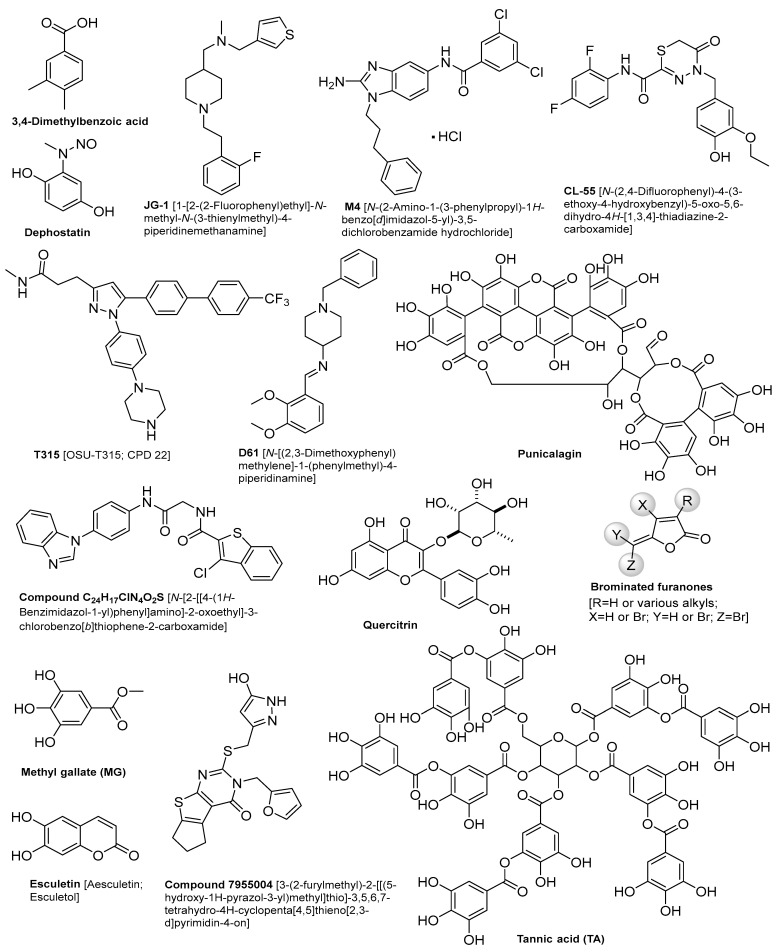
Summary of the chemical structures of compounds listed in Table 1 and Table 2.

Several SMs have shown promise in combating *Salmonella* infections, as illustrated in Table 1. The chemical structures of these compounds are described in Figure 1. A 3,4-dimethylbenzoic acid, which is an aromatic compound extracted from the human fecal microbiome, has shown great interference with gene expression involved in *Salmonella*’s invasion of host cells [30]. A combination of JG-1 and M4 small molecules has been utilized to inhibit biofilm formation and disrupt pre-existing biofilm structures of *S. typhi* in a mouse model of chronic gallbladder *Salmonella* carriage [31]. This treatment successfully prevented bacterial dissemination to peripheral organs [31]. Furthermore, a novel CL-55[*N*-(2,4-difluorophenyl)-4-(3-ethoxy-4-hydroxybenzyl)-5-oxo-5,6-dihydro-4H-[1,3,4]-thiadiazine-2-carboxamide] SM has demonstrated a significant reduction in *S. typhimurium* counts within the spleen and peritoneal lavages of mice [32]. Remarkably, complete eradication of *Salmonella* was achieved within twelve days of treatment [32]. Moreover, dephostatin, a non-antibiotic SM, has attenuated *S. typhimurium* virulence in mouse models and in vitro. It also restores sensitivity to polymyxin antibiotics [33]. Similarly, the T315 compound, when used in combination with ciprofloxacin, has demonstrated effective inhibition of biofilm formation in *S. typhimurium* and *S. typhi* serovars. It was reported that the half-maximal effective concentrations (EC_50_) were 7.4 μM for *S. typhimurium* and 21.0 μM for *S. typhi* [34]. Furthermore, D61 SMs have reduced *S. typhimurium* load in primary mice and human macrophages with no effect on *Salmonella* growth in epithelial cells. It has also reduced *Salmonella* load in the livers and spleens of infected mice [35]. Likewise, a previous study on four novel SMs has shown a significant growth inhibition of different *Salmonella enterica* serotypes in chickens and tomato [36,37]. They reduced the intracellular survival of *S. typhimurium* in eukaryotic models with a significant reduction of *S. typhimurium* load and colonization in chicken ceca and systemic organs [36]. Quercitrin, a flavonoid with antioxidant properties, has demonstrated significant inhibition of *S. typhimurium* adhesion, invasion, and survival in HeLa cell lines, reducing bacterial presence by 70% [38]. Moreover, Jacob et al., 2015 reported that compound 7955004 induced a 55% reduction in *S. typhimurium* preformed biofilms, achieving complete clearance of planktonic *S. typhimurium* in vitro [39].

However, SMs continue to play a pivotal role in modern therapeutics, they have limitations. Challenges such as poor bioavailability, off-target effects, and the potential for resistance, particularly in antimicrobial applications can hinder their effectiveness [40]. Moreover, their relatively simple structures may limit their ability to engage complex or large biological targets, where biologics often excel [41,42]. Despite these constraints, ongoing advances in medicinal chemistry and drug delivery technologies are steadily expanding the therapeutic potential SMs.

**Table 1:** Antimicrobial effect of SMs against different *Salmonella* spp. (for the chemical structures of the compounds, see Figure 1).

Small Molecules	Targeted *Salmonella* Serovars	Host	Observations	Refs.
3,4-dimethylbenzoic acid	*S. typhimurium*	In vitro	Treatment with 3,4-dimethylbenzoic acid led to a ~94% reduction in *hilA* expression. Furthermore, when epithelial cells were exposed to *Salmonella* treated with the compound, invasion rates dropped significantly, with statistical significance reported as *p* < 0.001 across three biological replicates. In addition, the bioactive fraction responsible for repression had a molecular weight, and retained activity comparable to the full fecal extract, showing ~79% repression of *hilA.*	[30]
JG-1 and M4	*S. typhi* and *S. typhimurium*	In vitro, Mice	Both compounds significantly reduced biofilm formation of *S. typhi* and *S. typhimurium* in vitro, with EC_50_ values of 38.9 μM (M4) and 53.6 μM (JG-1) for *S. typhi*. Additionally, M4 was more potent in dispersing pre-formed biofilms, with an EC_50_ of 46.4 μM, compared to 829 μM for JG-1. Also, in vivo treatment reduced bacterial burden in the gallbladder by 1–2 logs, and when co-administered with ciprofloxacin, the reduction reached 3–4.5 logs.	[31]
CL-55 (N-(2,4-difluorophenyl)-4-(3-ethoxy-4-hydroxybenzyl)-5-oxo-5,6-dihydro-4H-[1,3,4]-thiadiazine-2-carboxamide)	*S. typhimurium*	Mice	Mice treated with 10 mg/kg intraperitoneally for 4 days showed a 500-fold reduction in *S. typhimurium* counts in the spleen and peritoneal lavages. In addition, 12-day therapy led to the complete eradication of *Salmonella* from infected mice. Moreover, no detectable pathogen was found 4–6 weeks post-treatment, indicating durable clearance.	[43]
Dephostatin	*S. typhimurium*	In vitro, Mice	Dephostatin significantly reduced intracellular replication of *S. typhimurium* and restored susceptibility to colistin. Furthermore, mice infected with *S. typhimurium* and treated with Dephostatin and colistin showed prolonged survival compared to colistin alone, they also showed a >60% survival rate with combination therapy vs. 25% with colistin monotherapy.	[33]
T315	*S. typhi* and *S. typhimurium*	In vitro	T315 compound, when used in combination with ciprofloxacin, has demonstrated effective inhibition of biofilm formation in *S. typhimurium* and *S. typhi* serovars. Not only that, the half-maximal effective concentrations also (*EC_50_*) were determined to be 7.4 μM for *S. typhimurium* and 21.0 μM for *S. typhi*.	[34]
D61	*S. typhimurium*	In vitro, Mice	D61 significantly reduced *S. typhimurium* load in: RAW 264.7 macrophage-like cells (~20-fold reduction; IC_50_ = 1.3 μM), Primary mouse bone marrow-derived macrophages (~17-fold reduction; IC_50_ = 7.9 μM), and Primary human macrophages (~23-fold reduction). Also, D61 treatment led to a significant reduction in *Salmonella* burden in the spleen and liver of infected mice.	[35]
C_24_H_17_ClN_4_O_2_S	*S. typhimurium*	In vitro, chicken model	C_24_H_17_ClN_4_O_2_S inhibited the SPI-1 Type III Secretion System (T3SS) in *S. typhimurium*. In addition, it downregulated *InvF*, a key transcriptional regulator, and reduced effector proteins like *SipA* and *SipC*.	[36,44]
Quercitrin	*S. typhimurium*	In vitro, Mice	Quercitrin reduced *S. typhimurium* adhesion to HeLa cells in a dose-dependent manner, with no cytotoxicity observed up to 64 μg/mL. Moreover, in the mouse infection model, quercitrin treatment led to a ~1.5 log_10_ reduction in *S. typhimurium* counts in the cecal contents compared to untreated controls.	[38]
Compound 7955004	*S. typhimurium*	In vitro	7955004 induced a 55% reduction in preformed *S. typhimurium* biofilms. There was no toxicity observed in mammalian cells even at 30 μM, which is ~6× higher than EC_50_. Also, there was no significant change in OD_600_ of planktonic bacteria after 24 h exposure.	[39]

### 2.2. Quorum Sensing Inhibitors (QSIs)

Quorum sensing is a bacterial communication system that enables populations of bacteria to coordinate gene expression and behavior based on their density [45,46]. During growth, individual bacterial cells release signaling molecules called autoinducers into their environment [47]. As the population increases, so does the concentration of these molecules. Once a threshold level is reached, the autoinducers bind to specific receptors, triggering changes in gene expression that lead to collective behaviors such as biofilm formation, virulence factor production, and bioluminescence [45,47,48,49]. Once the concentration of the signaling molecule surpasses a specific threshold, it triggers the expression of certain genes in microorganisms, thereby affecting the pathogenicity and physiological processes of microorganisms [50]. Most quorum sensing inhibitors (QSIs) can disrupt the interaction between receptor proteins and signaling molecules, inhibiting the synthesis of autoinducers, or facilitating the degradation of quorum sensing signal molecules [51,52]. S-Ribosylhomocysteinase (*LuxS*) and Autoinducer 2-binding protein *LsrB* are two proteins involved in the quorum sensing system of *Salmonella*, playing crucial roles in modulating bacterial biofilm formation and pathogenicity [53,54,55]

Quorum sensing inhibitors (QSIs) represent a promising approach for controlling *Salmonella* infections by targeting key communication pathways, as illustrated in Table 2. For instance, brominated furanones are well-known quorum sensing inhibitors in various bacterial species, particularly *Pseudomonas aeruginosa*. However, their mechanism of action in *Salmonella enterica* remains unclear. No evidence that furanones interfere with the currently known quorum sensing systems in *Salmonella*, such as *SdiA* and AI-2 [56]. Nonetheless, brominated furanones have been shown to reduce biofilm formation and motility in *S. typhimurium*, suggesting they may exert indirect regulatory effects on virulence-associated pathways [56]. Treatment of *S. typhimurium* with methyl gallate (MG) at 30 µg/mL resulted in downregulation of quorum sensing genes *sdiA* (52.8%), *srgE* (61.7%), and rck (22.2%). When combined with a sub-minimum inhibitory concentration (sub-MIC) of marbofloxacin (MRB), MG further suppressed the host cell signaling gene rac-1 by 71.9%, compared to 56.9% with MG alone. Additionally, MG significantly reduced the expression of virulence-associated genes: *cheY* (59.6%), *ompD* (60.2%), *sipB* (20.5%), *lexA* (31.4%), and *ompF* (16.2%) [57]. Additionally, tannic acid was reported to inhibit the swarming motility of *S. typhi* and *S.* Paratyphi at a minimum effective concentration of 400 μg/mL, without affecting bacterial growth [58]. The synthetic compound *N*-(3-oxo-octanoyl)-DL-homoserine lactone at 10 nM has also been reported to suppress *sdiA* expression and inhibit biofilm development in *S. typhimurium* [59]. Xanthone derivatives have reduced AI-2 production by 60–70% in *S. typhimurium*, in conjunction with inhibition of efflux pump activity [60]. Moreover, punicalagin at a concentration of 15.6 μg/mL has demonstrated repressive effects on motility (*flhC*) and quorum sensing-related genes (*sdiA* and *srgE*) in *S. typhimurium* [61].

However, QSIs hold promise as a novel therapeutic approach to combat *Salmonella* infections in humans and animals; there are challenges remaining. Achieving species-specific targeting without disrupting the normal microbiota remains a significant challenge. Moreover, delivering effectively within complex environments such as biofilms poses additional obstacles, particularly in terms of ensuring their bioavailability. Furthermore, the high costs and extended timelines associated with QSIs development limit their widespread application as therapeutic agents.

**Table 2:** Antimicrobial effect of QSIs against different *Salmonella* spp. (For the chemical structures of the compounds, see Figure 1).

QSIs	Targeted Salmonella Serovars	Host	Observations	Refs.
Brominated Furanones	*S. typhimurium*	In vitro	Brominated furanones do not act on known QS systems in *Salmonella* (e.g., *SdiA*, AI-2) but may exert indirect effects on biofilm and motility.	[56]
Punicalagin	*S. typhimurium* SL1344	In vitro	Punicalagin, even at sub-inhibitory concentrations (1/16× and 1/32× MIC), significantly reduced *Salmonella* motility, including both swimming and swarming behaviors. This reduction was associated with the downregulation of key motility-related genes (*fliA*, *fliY*, *fljB*, *flhC*, and *fimD*). Additionally, punicalagin dose-dependently inhibited violacein production in *Chromobacterium violaceum*, indicating its quorum sensing inhibitory activity. In *Salmonella*, it suppressed the expression of QS-regulated genes (*sdiA* and *srgE*) and significantly reduced bacterial invasion of human colonic cells (*p* < 0.01) without affecting adhesion.	[61]
Methyl gallate (MG)	*S. typhimurium*	In vitro	Exposure of *S. typhimurium* to methyl gallate at a concentration of 30 µg/mL led to notable downregulation of quorum sensing genes, including *sdiA* (52.8%), *srgE* (61.7%), and *rck* (22.2%). When MG was combined with a sub-minimum inhibitory concentration of marbofloxacin, suppression of the host cell signaling gene *rac-1* increased to 71.9%, compared to 56.9% with MG alone. Furthermore, MG significantly reduced the expression of key virulence-associated genes: *cheY* (59.6%), *ompD* (60.2%), *sipB* (20.5%), *lexA* (31.4%), and *ompF* (16.2%).	[62]
Tannic acid	*S. typhi*, *S.* Paratyphi	In vitro	Tannic acid exhibited strong quorum sensing inhibitory activity at a minimum effective concentration of 400 μg/mL. It reduced cell surface hydrophobicity by 38–43% and extracellular polymeric substance (EPS) production by 35–50%. TA significantly enhanced the susceptibility of *S. typhi* and *S.* Paratyphi to a range of antibiotics, including amikacin, ampicillin, ciprofloxacin, azithromycin, chloramphenicol, and gentamicin. Additionally, TA drastically inhibited swarming motility, a key quorum sensing-regulated phenotype, without affecting bacterial growth.	[58,63]
Star anise extract	*S. typhimurium*	Food matrix	Star anise extract inhibited violacein production by 89% in *Chromobacterium violaceum*, indicating strong interference with quorum sensing-regulated pigment synthesis. Although this biosensor assay does not directly evaluate quorum sensing in *S. typhimurium*, it suggests that the extract may possess broad-spectrum quorum sensing inhibitory activity. Supporting this, swarming motility in *S. typhimurium* was significantly reduced—by up to 95.9%—at higher extract concentrations, highlighting its potential to impair bacterial communication and motility.	[64]
Esculetin	*S. typhimurium*	Chicken	Esculetin demonstrated strong antimicrobial and quorum sensing inhibitory activity against *S. typhimurium*, with MIC of 500 μg/mL. It downregulated key genes involved in quorum sensing and biofilm regulation, including *adrA*, *lsrB*, *luxS*, and *rpoS*. Mechanistically, esculetin competes with the *LsrB* receptor, preventing *AI*-2 uptake and disrupting QS signaling. At 8× MIC, esculetin was able to kill 2 log CFU/mL of *S. typhimurium* within 30 min.	[53]

### 2.3. Probiotics

**Figure 2:**
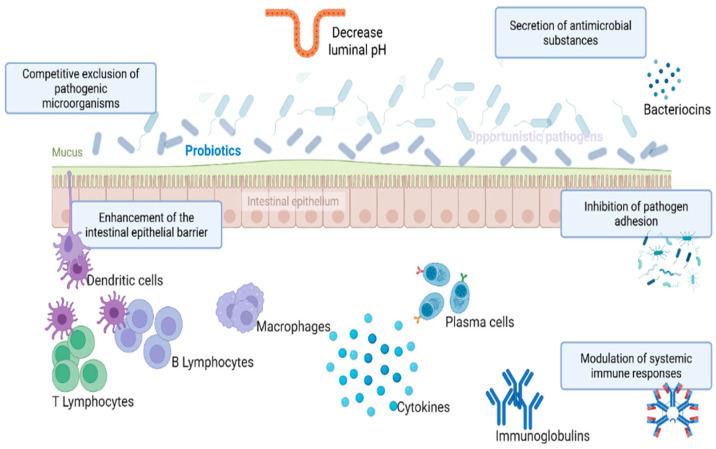
Mechanism of action of probiotics. These include: (1) Competitive exclusion of pathogenic microorganisms via adhesion to epithelial surfaces and occupation of receptor sites; (2) Secretion of antimicrobial substances such as bacteriocins, organic acids, and hydrogen peroxide that inhibit pathogen growth; (3) Modulation of host immune responses, including enhancement of mucosal immunity, stimulation of secretory IgA production, and activation of innate immune cells; (4) Competition for nutrients and ecological niches, limiting the availability of resources for pathogenic species; (5) Strengthening of intestinal barrier integrity by promoting tight junction protein expression and reducing epithelial permeability.

Probiotics are live, non-pathogenic microorganisms that, when administered in adequate amounts, enhance gut health and confer a health benefit on the host [65,66,67]. The selection of probiotic strains for potential therapeutic use, particularly as antimicrobial alternatives, is guided by specific criteria. These criteria include the capacity to withstand the challenging conditions of the gastrointestinal tract, such as low pH and bile salts, the demonstrated safety for host consumption, the proven efficacy against targeted pathogenic bacteria, the ability to efficiently colonize the intestinal epithelium, and the maintenance of genotypic and phenotypic stability [29,68]. Probiotics could exert their beneficial effects through different mechanisms of action, as illustrated in Figure 2. They can adhere to the gut mucosa and exclude pathogenic bacteria from adhesion sites in the gut. They strengthen the gut lining and mucosal integrity, preventing harmful substances and toxins from invading the bloodstream. They maintain a healthy balance of beneficial gut microbiota, as well as increasing the bioavailability and absorption of micro- and macro-nutrients. Probiotics can also produce antimicrobial compounds, such as bacteriocins, which suppress the growth of pathogenic microorganisms. Additionally, probiotics interact with intestinal epithelial cells, reducing the production of anti-inflammatory cytokines and facilitating the recruitment of mononuclear cells and macrophages [69,70,71,72]. Probiotics can be categorized into three types: single-strain probiotics, which contain only one bacterial strain; multi-strain probiotics, which include multiple strains of the same species; and multi-species probiotics, which consist of strains from different bacterial species combined [73,74]. The combination of multiple groups of probiotics tends to have a synergistic effect on each other as well as broaden the spectrum of activity against different pathogens [75]. Numerous studies have shown that specific probiotic strains from the genera *Lactobacillus*, *Bifidobacterium*, *Streptococcus*, *Pediococcus*, *Enterococcus*, and *Bacillus* possess the ability to suppress the growth and virulence of various pathogens, including *Salmonella,* as illustrated in Table 3. Despite the promising value and potential benefits of probiotics for controlling and treating *Salmonella* infection, their clinical application still faces several challenges. These include the ability of probiotics to withstand bile salts and gastric juice in the gastrointestinal (GI) tract [76,77], the risk of acquiring resistance genes from pathogenic microorganisms [78,79], and the potential for certain probiotic strains to produce toxic substances that may induce alimentary infection and intoxication [80,81,82], as well as safety concerns for immunocompromised individuals and those with underlying health conditions.

### 2.4. Prebiotics

Prebiotics are non-digestible compounds that can promote the growth of beneficial bacteria. Prebiotics are often paired with probiotics to form synbiotics, a synergistic combination that significantly enhances therapeutic effectiveness against a range of pathogens [114]. There are different available prebiotics such as galacto-oligosaccharides (GOS), fructo-oligosaccharides (FOS), mannan-oligosaccharides (MOS), trans-galacto-oligosaccharides (TOS), xylo-oligosaccharides (XOS), arabinoxylo-oligosaccharides (AXOS), inulin, and lactulose [115,116]. These prebiotics are normally not digested by digestive enzymes, allowing them to stimulate the growth and activity of gut microbiota [72,117]. Prebiotics should have certain criteria to be effectively applied, such as resistance to the acidic environment of the gastrointestinal tract, susceptibility to fermentation by the gut microbiota, and the ability to selectively promote the activity and/or growth of beneficial bacterial populations [118]. The fermentation of prebiotics by gut microbiota leads to the production of short-chain fatty acids (SCFAs), including butyric acid, propionic acid, and lactic acid, which all play a crucial role in maintaining intestinal health and metabolic functions [115]. Short-chain fatty acids, produced through the fermentation of prebiotics by gut microbiota, can positively impact the host by stimulating the innate immune response and enhancing defense mechanisms against pathogenic microorganisms [119]. SCFAs can also influence the intestinal epithelial cell development [120]. Beyond their local effects within the gastrointestinal tract, the beneficial actions of prebiotics can extend to distant organs and systems, facilitated by the systemic bioavailability of their fermentation products, specifically SCFAs. Prebiotics have shown promising results in combating *Salmonella* infections in vitro and in vivo, particularly when combined with probiotics. It has been demonstrated that certain prebiotics inhibit the growth of *Salmonella* through direct or indirect stimulation of beneficial gut microbiota. Micciche et al., 2018 highlighted how oligosaccharides stimulate SCFA production, bolster microbial balance, and reduce pathogen load in poultry [121]. Additionally, Ismael et al., 2025 emphasized that prebiotics contribute to colonization resistance against drug-resistant *Salmonella* via microbial competition and immune modulation [122]. Khan et al., 2020 showed that compounds like inulin and galacto-oligosaccharides (GOS) enhance microbial diversity and strengthen mucosal barriers [65], while Deng and Wang reviewed how dietary fibers foster antimicrobial metabolite production and inhibit *Salmonella* through nutrient competition and quorum sensing disruption [123]. These beneficial bacteria can produce certain substances that suppress *Salmonella* growth and reduce its colonization in the gut [64]. Despite the beneficial effect of prebiotics to control *Salmonella* infection as illustrated in Table 4, there are some studies that have shown that certain prebiotics might even increase the severity of *Salmonella* infections under specific circumstances, potentially by impairing the gut barrier function or promoting the growth of certain pathogenic microorganisms [124]. For example, a study on mice fed diets with 10% FOS or XOS showed a significantly higher *S. typhimurium* count in the liver, spleen, and lymph nodes (*p* < 0.01) compared to controls [125].

### 2.5. Postbiotics

Postbiotics are bioactive compounds derived from microbial metabolism, including metabolites produced during fermentation, components released from inactivated (non-viable) microbial cells, and other microbial byproducts [134,135]. Unlike probiotics, which are live bacteria, postbiotics are beneficial byproducts of microbial activity. These compounds include short-chain fatty acids, enzymes, vitamins, and antimicrobial peptides, all of which contribute to gut health and overall well-being [136,137]. One of the most significant advantages of postbiotics is their role in immune system regulation. Certain postbiotics, such as butyrate, help stimulate the production of regulatory T cells, which control immune responses and reduce inflammation [138]. Additionally, postbiotics can enhance the gut barrier, preventing harmful bacteria from entering the bloodstream [139,140]. Since postbiotics are the final product of probiotic activity, they offer a safe and effective way to support gut health without the risks associated with live bacterial supplementation [137]. Importantly, postbiotics offer enhanced safety and stability, making them easier to handle and store compared to live probiotics [141]. Their inanimate nature eliminates concerns about viability, ensuring consistent effectiveness in various applications.

Postbiotics have demonstrated promising antimicrobial effects against *Salmonella*, particularly in applications related to food safety and gut health. Certain lactic acid bacteria-derived postbiotics, such as *Lactobacillus*, *Bifidobacterium*, and *Pediococcus* species, can inhibit the growth of *Salmonella* by producing a variety of bioactive compounds, such as SCFAs, bacteriocins and antimicrobial peptides [142,143,144]. These compounds help create an unfavorable environment for *Salmonella*, reducing its ability to colonize and cause infections. For example, Yin et al. found that weaned pigs fed on *Lactobacillus*-fermented feed have shown a significant reduction in *S. typhimurium* count by 50% in the spleens of pigs [145]. Additionally, Harris et al. investigated the effect of *Saccharomyces cerevisiae* fermentation products on the overall health performance of pre-weaned calves challenged with *S. typhimurium* [146]. They found that there was an improvement in fecal consistency and the immune status of the challenged calves [146]. Moreover, postbiotics have been explored as a natural intervention for controlling *Salmonella* contamination in poultry and eggs, offering an alternative to traditional disinfectants [147,148]. As research continues to validate their safety, stability, and efficacy, postbiotics stand poised to become a cornerstone of next-generation strategies for promoting gut health, enhancing immunity, and combating foodborne pathogens like *Salmonella*.

### 2.6. Antimicrobial Peptides (AMPs)

AMPs are emerging as a powerful alternative to conventional antibiotics, especially in the fight against MDR bacterial infections [149]. AMPs, typically ranging from 12 to 60 amino acids in length, are endogenous molecules present on mucosal surfaces and within tissues across diverse biological kingdoms, including humans, animals, and plants [150]. As integral components of the innate immune system, they exert broad-spectrum antimicrobial effects through multiple mechanisms of action [151,152].

AMPs have two primary mechanisms; (a) Membrane targeting mechanism through which AMPs may either align parallel to the cell membrane like a carpet (carpet-like model), or aggregate with each other and form channels that cause cytoplasmic leakage (barrel-stave model), or form a ring hole by vertically embedding in the bacterial cell membrane (toroidal pore model) [153,154,155]. (b) Non-membrane targeting mechanism through which AMPs target nucleic acid biosynthesis, protein biosynthesis, metabolic activities, nucleic acid replication and cell division [156,157]. There are many examples of AMPs that can ameliorate foodborne pathogens, including *Salmonella*. For example, AP2, an optimized version of native apidaecin (AP IB), has a protective effect against *S. typhimurium* infection in mice [158]. It ameliorated *S. typhimurium* infection by modulating the gut microbiota [158]. Another example is the 1018-K6 innate defense peptide that has been evaluated against *S. enterica*. It revealed a strong biofilm inhibition of several *S. enterica* strains using the sub-inhibitory concentrations [159]. Furthermore, antimicrobial peptides such as Kn2-5R-NH2, WK2, JH-3 and Bac7 (1–35) have been investigated a significant in vivo bactericidal effect against MDR-*Salmonella* strains [160,161,162,163]. Similarly, a preliminary in vivo study on WK2, a *β*-hairpin peptide, has demonstrated a significant reduction in *S. typhimurium* in the liver and spleen with inflammation relief and maintenance of intestinal mucosal integrity in mice [161]. Moreover, the thermostable modified cathelicidin-derived peptide P7 has demonstrated a significant antibacterial effect by reducing *S. typhimurium* viable cell counts to 10^3^ and 10^4^ CFU/mL within 2 to 4 h, respectively [164]. Mechanistically, P7 exerts its bactericidal effect by binding to the bacterial membrane, penetrating it, and accumulating within the cytoplasm. This interaction leads to membrane depolarization, permeabilization, and subsequent leakage of intracellular contents, culminating in rapid bacterial cell death [164]. Notably, a combination of a novel AMP derivative (A11) and nisin had a synergistic effect on drug-resistant *S. typhimurium* strains in vitro [165]. A11 destabilizes the bacterial membrane, increasing permeability, while Nisin binds to lipid II and forms pores, accelerating cell death. This dual action amplifies bacterial membrane damage, leading to a greater reduction in viable cell counts than either compound alone [165].

Another study on antimicrobial peptide HJH-3 has explored the significant antimicrobial effect against *S. pullorum* at higher peptide concentration [166]. The increased concentrations of HJH-3 led to a dose-dependent reduction in *S. pullorum* viability, indicating strong bactericidal activity [166]. Furthermore, a murine cathelicidin-related antimicrobial peptide (CRAMP) has increased macrophage expression and impaired *S. typhimurium* division and replication in vitro and in vivo by using an oxidase-dependent mechanism [167]. Additionally, the antimicrobial peptide Microcin J25 (MccJ25) has demonstrated significant efficacy in reducing *Salmonella* CVCC519 infection rates in broiler chickens. On day 42, challenged birds exhibited a 30% reduction in infection compared to the untreated control group [168]. Moreover, complete inhibition and elimination of *S. typhimurium* was observed in challenged mice treated with C-terminally hexahistidine-tagged A3-APO, loaded onto a gold nanoparticle DNA conjugate [169]. In addition, the antimicrobial peptide Css54 exhibits a potent growth inhibitory effect on *S. typhimurium* at a concentration of 6.25 μg/mL, with complete bactericidal activity achieved at 25 μg/mL [170].

Despite AMPs having proven efficacy in targeting bacterial pathogens, concerns remain regarding bacterial adaptation and resistance development. Some bacteria can modify their membrane composition or employ efflux pumps to counteract AMPs, reducing their efficacy over time. Additionally, certain peptides may inadvertently affect mammalian cells, leading to cytotoxic effects. So, optimizing AMP design through structural modifications and targeted delivery systems can help mitigate these challenges and enhance their therapeutic potential [153,154,155].

### 2.7. Essential Oils (EOs)

Essential oils consist of a complex mixture of volatile compounds that demonstrate considerable promise in biomedical applications. These compounds are derived from various plant parts, including leaves, flowers, stems, roots, bark, buds, and wood [171]. They exhibit potent antimicrobial properties and effectively suppress the growth of various bacteria, fungi, and viruses. Their efficacy is largely influenced by their chemical composition, the nature of their bioactive compounds, and the spatial orientation of their functional groups [172]. EOs can exhibit antimicrobial activity in its vapor phase without requiring direct contact with the pathogen [173].

EOs can be extracted using a range of techniques, such as steam distillation, solvent extraction, fermentation, and effleurage. The choice of method significantly affects the purity, chemical composition, and yield of the oil, thereby determining its suitability for specific plant materials and intended applications [174]. EOs demonstrates enhanced bioactivity when they exist in their active or oxygenated forms, as these molecular structures optimize their interactions with microbial cells, contributing to its antimicrobial, antioxidant, and therapeutic properties [175]. The antimicrobial activity of EOs is mediated through multiple mechanisms, including inhibition of biofilm formation, disruption of cell membrane integrity and permeability, interference with quorum-sensing pathways, suppression of bacterial protein synthesis, and impairment of cellular energy production [176,177,178]. These multifaceted actions contribute to their broad-spectrum antimicrobial potential [73,74]. Furthermore, the antimicrobial effectiveness of essential oils is influenced by the specific microbial species targeted, as different pathogens exhibit varying susceptibilities to the bioactive compounds within EOs [179,180]. Other factors, such as microbial cell structure, resistance mechanisms, and metabolic pathways, play a role in determining the extent of inhibition [181].

Essential oils are frequently utilized as natural preservatives in the food industry to inhibit the proliferation of foodborne pathogens, including *E. coli*, *Salmonella*, *Listeria monocytogenes*, and *Campylobacter* [178,182]. Their antimicrobial properties contribute to food safety by reducing contamination risks and extending the shelf life of products [183]. EOs derived from thyme, rosemary, oregano, clove, cinnamon, and curcuma are among the most widely recognized natural agents for combating antibiotic-resistant *Salmonella* and other pathogenic microorganisms [184]. Different studies evaluated the synergistic antimicrobial effect of combined EOs against *Salmonella,* as shown in Table 5.

While EOs have demonstrated significant antimicrobial activity against *Salmonella* spp., including *S. typhimurium* and *S. enteritidis*, their application requires careful consideration due to potential safety concerns. The high potency of EOs, particularly those from oregano, thyme, cinnamon, and clove, can lead to cytotoxic effects if not properly diluted, especially when used in food systems or biomedical formulations [185]. Additionally, variability in chemical composition due to plant origin, extraction method, and storage conditions can affect both efficacy and safety. Also, some EOs may also interact with host microbiota or immune responses, so their use in vulnerable populations such as young or immunocompromised hosts should be approached with caution.

**Table 5:** Antimicrobial effect of essential oils against different *Salmonella* spp.

Essential Oils, EOs	*Salmonella* Serovars	Host	Observations	Ref.
EOs from leaves of *Coriandrum sativum* L.	*S. typhi*	In vitro	The oil exhibited strong antibacterial and antifungal activity against all tested strains, such as *Staphylococcus aureus*, *Bacillus* spp., *E. coli*, *S. typhi*, *Klebsiella pneumoniae*, *Proteus mirabilis* and *Candida albicans*, except *Pseudomonas aeruginosa*, which was resistant. Notably, a 65 × 10^2^ μg concentration of EO resulted in a zone of inhibition (13.0 ± 1.4 mm) against *S. typhi*.	[186]
A blend of thyme EOs, savory, peppermint, and black pepper seeds	*S. enteritidis*	Broiler chickens	A microencapsulated blend of thyme essential oil (50%), savory (25%), peppermint (12.5%), and black pepper seeds (12.5%) caused a significant decrease in *S. enteritidis* population in broiler chickens. Specifically, the bacterial load in the ileum decreased by 2.1 log_10_ CFU/g, and in the cecum by 2.4 log_10_ CFU/g compared to the untreated infected group (*p* < 0.05).	[187]
EOs derived from the seeds of *Foeniculum vulgar* and *Cuminum cyminum* L.	*S. typhimurium*, *E. coli*	In vitro	*F. vulgare* and *C. cyminum* EOs induced zones of inhibition measuring 33 and 22 mm against *S. typhimurium*, respectively. In addition, the minimum inhibitory concentrations (MICs) were determined to be 0.031 mg/mL for *F. vulgare* and 0.125 mg/mL for *C. cyminum.* Furthermore, *F. vulgare* and *C. cyminum* EOs induced zones of inhibition measuring 28 and 17 mm against *E. coli*, respectively. In addition, the minimum inhibitory concentrations (MICs) were determined to be 0.062 mg/mL for *F. vulgare* and 0.250 mg/mL for *C. cyminum*	[188]
Carvacrol, eucalyptol, thymol and lemon EO blend	*S. heidelberg*	Broiler chickens	At a concentration of 0.05%, EOs significantly (*p* < 0.05) reduced *S. heidelberg* colonization in the crop of infected broilers. Additionally, lower concentrations (0.025% and 0.015%) showed no significant effect. However, EOs did not impact colonization in the ceca or fecal shedding, indicating their antimicrobial activity may be localized to specific regions of the gastrointestinal tract.	[189]
Zahter extract, zahter essential oil, laurel extract, and laurel essential oil	*S. typhimurium*	Chicken wings	The 0.4% laurel exhibited the strongest inhibitory effect against *S. typhimurium*, while the zahter showed comparatively lower antimicrobial activity. These findings suggest that laurel may possess superior bioactive compounds for bacterial suppression.	[190]
*Satureja hortensis*	*S. enteritidis*	In vitro	The disc diffusion assay showed that *Salmonella* had an average inhibition zone of 38 mm with a standard deviation of ±4 mm. in addition, both half and quarter concentrations of the MIC/2 and MIC/4 effectively suppressed biofilm formation by *S. enteritidis.*	[191]
Blend of cinnamaldehyde, thymol, citral, carvacrol, β-pinene and limonene	*S. enteritidis*	Chicken	Thymol, carvacrol, and cinnamaldehyde significantly inhibited *S. enteritidis* biofilm formation in a concentration-dependent manner. Thymol at MIC reduced biofilm formation as early as 12 h (*p* < 0.05), with stronger effects observed at 24 and 48 h. Carvacrol at MIC also showed consistent inhibition across all time points (*p* < 0.05), though lower concentrations (1/2 and 1/4 MIC) unexpectedly promoted biofilm formation at 12 and 24 h (*p* < 0.05). Cinnamaldehyde significantly reduced biofilm biomass at all concentrations after 24 and 48 h (*p* < 0.05).	[192]
EOs blend derived from *Origanum vulgare, Thymus serpyllum, Thymus vulgaris*, and *Melaleuca alternifolia*	25 MDR *Salmonella* strains	In vitro	EOs of *T. serpyllum* and *O. vulgare* showed a significant antimicrobial activity against MDR-*Salmonella* strains.	[193]
Cinnamaldehyde, carvacrol, thymol, eugenol and citral	*S. typhimurium*, *C. jejuni*	In vitro	All tested compounds demonstrated strong bactericidal activity. The lowest concentration of trans-cinnamaldehyde (10 mM) significantly reduced (*p* ≤ 0.05) *S. enteritidis* populations by approximately 6.0 log_10_ CFU/mL after 8 h, and by more than 8.0 log_10_ CFU/mL after 24 h of incubation. At a concentration of 25 mM, trans-cinnamaldehyde eliminated detectable (*p* ≤ 0.05) *S. enteritidis* within 8 h. Furthermore, carvacrol and eugenol also significantly decreased (*p* ≤ 0.05) populations of *S. enteritidis* and *C. jejuni* to below 1.0 log_10_ CFU/mL at concentrations of 50 and 75 mM for carvacrol, and 20 and 30 mM for eugenol.	[194]

### 2.8. Organic Acids (OAs)

Organic acids are carbon-based molecules composed of short- and medium-chain fatty acids. The chemical structure and acidic properties of OAs are essential for their antimicrobial and antifungal efficacy. OAs are categorized within the fatty acid classification and encompass a range of compounds, including acetic acid, lactic acid, formic acid, propionic acid, sorbic acid, butyric acid, benzoic acid, and citric acid [195]. These acids play essential roles in biochemical processes, food preservation, and antimicrobial applications. OAs can disrupt bacterial metabolic activities through lowering the intracellular pH, altering the bacterial cell membrane permeability, interfering with the bacterial energy production pathways, and interfering with bacterial DNA replication and transcription processes [73].

Studies have demonstrated that bacterial strains exhibit distinct sensitivity to various OAs, which can significantly influence their antimicrobial effectiveness. For instance, *Salmonella enterica* is particularly susceptible to formic and propionic acids, which disrupt membrane integrity and lower intracellular pH [196]. *E. coli* O157:H7 shows greater sensitivity to acetic acid than lactic acid, due to acetic acid’s superior ability to penetrate the cell membrane and acidify the cytoplasm [196]. Additionally, *L. monocytogenes* is effectively inhibited by lactic acid and citric acid, which interfere with its energy metabolism and membrane function [196]. *Campylobacter jejuni* is highly sensitive to medium-chain fatty acids like butyric and caprylic acids, which compromise membrane potential and ATP synthesis [197]. Meanwhile, *Clostridium perfringens* is notably affected by sorbic and benzoic acids, which hinder spore germination and vegetative growth [197]. These differences underscore the importance of selecting specific organic acids based on the target bacterial strain for optimal antimicrobial efficacy. Several studies have indicated that multi-chain fatty acids have more potent antimicrobial effects compared to short-chain fatty acids [198]. Furthermore, using organic acid blends provides a broader spectrum of activity than a single organic acid at a higher dose [199].

There are mixtures of different OAs that showed great efficacy in reducing the load of viable *Salmonella* bacterial cells in various animal species and food products [187]. For example, the organic acid blend, consisting of 0.024% tannic acid, 0.042% lactic acid, 0.048% butyric acid, and 0.048% acetic acid, demonstrated efficacy in reducing *S. enteritidis* levels by at least 1 log in broiler chicken (*p* = 0.05) [200]. This reduction suggests the potential of OAs as a microbial control strategy in poultry production. In addition, it had a significant effect in reducing *S. enteritidis* horizontal transmission among broiler chickens, whether administered solely or in combination with other probiotics (*p* = 0.05) [200]. Notably, pigs fed on OAs combined with Mannan-rich Copra Meal (OA/MCM) and OAs combined with Fermented Rye (OA/FR) diets exhibited significantly reduced *S. typhimurium* shedding post-infection (*p* < 0.05) [201]. Moreover, broiler chickens fed a blend of three different OAs (Sorbic acid, 25%; Thymol, 9.5%; Carvacrol, 2.5%) showed a reduction in *S. heidelberg* shedding and counts compared to the control birds [202]. Furthermore, administering esterified formic acid at 10 kg per 1000 L in water for five days before pig’s slaughter led to a 60% reduction in the proportion of *Salmonella*-shedding in pigs. Additionally, the odds of shedding *Salmonella* were 5.6 times higher in untreated pigs compared to those receiving treatment [203]. Another study comparing the efficacy of different OAs against *S. enteritidis* at the same concentration showed that capric acids and caprylic acids possessed bactericidal properties and eliminated *S. enteritidis* in vitro compared to other organic acids such as citric acid, fumaric acid, benzoic acid and acetic acid [204]. Moreover, a study reported a substantial reduction up to 2.5 log units in the bacterial counts of *S. typhimurium*, *S. infantis*, *S. senftenberg*, and *S. putten* when mash and rapeseed-based feed were supplemented with propionic acid, formic acid, and sodium format [205].

Despite their proven antimicrobial properties, the use of OAs in feed and water systems presents several limitations. One major challenge is their limited efficacy in the lower gastrointestinal tract, where *Salmonella* colonization typically occurs. This is due to the early absorption and metabolism of OAs in the upper digestive tract, which reduces their concentration at the site of infection. Additionally, high inclusion rates required for effective pathogen control can negatively impact feed palatability and animal performance and may even corrode feeding equipment. Moreover, OAs are primarily bacteriostatic, meaning they inhibit bacterial growth rather than eliminate pathogens, leaving room for recontamination during storage and transport. Finally, strain-specific resistance and the potential for microbial adaptation to acidic environments further complicate their long-term effectiveness.

### 2.9. Vaccines

Vaccines are biological preparations designed to stimulate the body’s immune system to recognize and combat specific infectious agents, such as viruses or bacteria [206]. They typically contain either attenuated (weakened) or inactivated forms of the pathogen, or purified components such as proteins, polysaccharides, or genetic material (e.g., mRNA or DNA) [207,208]. These elements serve as antigens that trigger an immune response, enabling the body to recognize and respond more effectively to future exposures to the actual pathogen [209,210]. Most vaccines do not completely prevent infection, but they are highly effective at reducing disease severity and complications. Additionally, they contribute to lowering the likelihood of disease transmission between hosts [211,212,213]. Their classification governs their mechanism of action. Live attenuated vaccines use a weakened form of a live virus or bacteria to stimulate the immune system and provide protection against a specific disease. They can stimulate humoral immunity and activate cytotoxic T-cells, resulting in long-lasting immunity [214,215]. Inactivated vaccines contain killed pathogens, so they cannot replicate or cause disease. They induce immunity without the risk of infection and may require multiple doses compared to live vaccines [216]. Subunit vaccines containing a specific part (proteins, polysaccharides, nucleic acids) of the pathogen, stimulating an immune response instead of the entire pathogen [217]. Additionally, conjugate vaccines are a combination of polysaccharide and a protein carrier. They combine the immunogenicity of protein carriers and the specificity of polysaccharides, providing effective and long-lasting protection [218]. Toxoid vaccines contain inactivated bacterial toxins that stimulate the immune response against specific diseases [219]. Outer Membrane Vesicle (OMV) vaccines consist of naturally released vesicular components derived from the bacterial outer membrane. These vesicles effectively stimulate an immune response while avoiding the induction of disease, making them a promising strategy for vaccine development [220]. Nucleic acid vaccines use genetic material (mRNA or DNA) to enhance cells to produce antigens and trigger the immune response [221]. Nanovaccines are a novel vaccine platform that utilizes nanoparticles (NPs) as adjuvants or delivery carriers. This approach facilitates precise targeting of infection sites while simultaneously enhancing systemic immune responses, improving vaccine efficacy and stability [222]. There are many examples of vaccines designed to target *Salmonella* in both humans and animals, as shown in Table 6. Despite the progressive development of vaccines targeting *Salmonella*. There are still different challenges include the extensive genetic diversity between *Salmonella* serovars [223], the potential for tolerance to toxoid vaccines, and the relatively low immunogenicity observed in outer membrane vesicle (OMV) vaccines [217,224].

### 2.10. Bacteriophages

**Figure 3:**
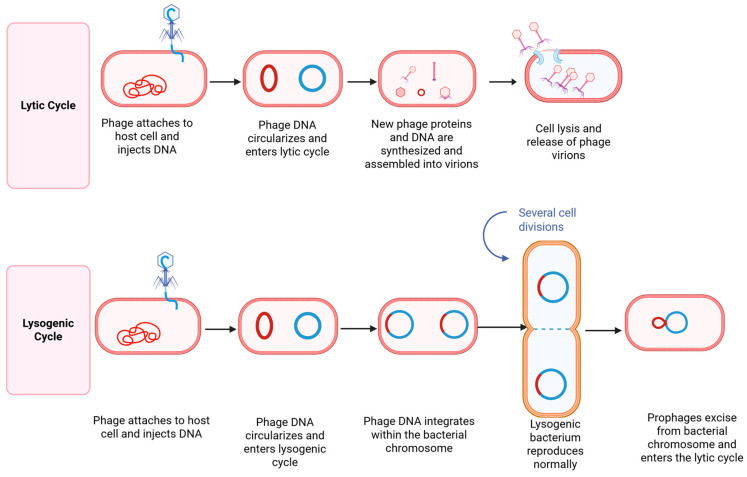
Mechanism of action of bacteriophages via lytic and lysogenic cycles. Created with BioRender.com. In the lytic cycle, the phage attaches to the bacterial surface and injects its nucleic acid into the host. The viral genome commandeers the host’s replication machinery to synthesize phage components, assemble new virions, and produce lytic enzymes such as endolysins. These enzymes degrade the bacterial cell wall, resulting in cell lysis and the release of progeny phages to infect neighboring cells. In the lysogenic cycle, the phage genome integrates into the bacterial chromosome, forming a dormant prophage. This prophage replicates passively with the host genome during cell division.

Bacteriophages are viruses that selectively infect and lyse bacterial cells, functioning as highly host-specific bacterial parasites. Due to their targeted antimicrobial activity, they are increasingly recognized as a promising alternative to conventional antimicrobials, contributing to enhanced food safety and security. This diversity in form and function underpins their versatility in biotechnology, diagnostics, and antimicrobial therapy. Bacteriophages can specifically infect and lyse bacterial cells through a highly targeted process, as illustrated in Figure 3. Upon encountering a susceptible host, the phage attaches to bacterial surface receptors using tail fibers or other specialized structures. It then injects its genetic material, either DNA or RNA into the bacterial cytoplasm, hijacking the host’s replication machinery to produce viral components [236]. In the lytic cycle, newly assembled phage particles accumulate until the host cell undergoes lysis, releasing progeny phages to infect neighboring bacteria. Alternatively, in the lysogenic cycle, the phage genome integrates into the host chromosome and replicates passively until triggered to enter the lytic phase [236,237]. For therapeutic purposes, only strictly lytic phages are considered suitable, as temperate phages capable of lysogeny may contribute to horizontal gene transfer and bacterial persistence, making them undesirable in clinical applications.

Several factors influence the therapeutic efficacy of bacteriophages in treating bacterial infections. One key determinant is the phage host specificity, as phages must precisely match their bacterial targets to be effective. The timing and mode of administration, whether oral, topical, or intravenous, also impact outcomes, as does the multiplicity of infection (MOI), or the ratio of phages to bacteria. Environmental conditions such as pH, temperature, and the presence of biofilms can hinder phage access or activity. Additionally, immune system interactions may neutralize phages before they reach their targets, and bacterial resistance mechanisms can emerge, reducing long-term effectiveness. Other considerations include phage stability, distribution within the host, and the site of infection, all of which must be optimized for successful therapy [238,239]. Several studies identified bacteriophage cocktails as a promising tool against MDR pathogens, including *Salmonella.* A *Salmonella*-infected *Galleria mellonella* larva model was able to survive, and the *Salmonella* serovars including *S. typhimurium*, *S. enteritidis*, *S. infantis*, *S. agona*, *S. derby*, *S. ohio*, *S. brandenburg* and *S. rissen* were undetectable 24 h post administration of the three-phage cocktail [240]. Furthermore, the protective efficacy of *Salmonella*-specific bacteriophages and competitive exclusion products was assessed in chickens experimentally infected with *S. typhimurium*. Treatments with bacteriophages, competitive exclusion products, and their combination resulted in a marked reduction in *S. typhimurium* to undetectable levels in both the ileum and cecum, compared to untreated controls [241]. Similarly, an aerosol spray containing three distinct lytic bacteriophages, isolated from the sewage system of chicken flocks, demonstrated a significant reduction (72.7%) in the incidence of *S. enteritidis* (SE) infection within the challenged group compared to the control group [242]. Additionally, phage administration via drinking water or coarse spray effectively lowered intestinal *S. enteritidis* colonization, with statistically significant reductions (*p* < 0.05; *p* < 0.01, respectively) [242]. A higher *Salmonella* reduction was also observed after the spread of commercially available *Salmonella* bacteriophage on *Salmonella*-free skinless, boneless chicken meat inoculated with a cocktail of three *Salmonella* isolates [243]. Moreover, the bacteriophage cocktail SalmoFREE^®^ has demonstrated effectiveness in significantly reducing the incidence of *S. enteritidis* in a commercial broiler farm. Importantly, this intervention did not negatively impact the production parameters of broiler chickens [244]. In experimentally contaminated feed, BAFASAL^®^ reduced *S. enteritidis* counts by up to 2.5 log_10_ CFU/g under simulated crop conditions [245]. In in vivo trials, broiler chickens treated with BAFASAL^®^ showed a significant reduction in *S. enteritidis* in the caeca, with bacterial counts dropping by approximately 1.5 to 2 log_10_ CFU/g compared to untreated controls [245]. Importantly, no signs of toxicity were observed, even at doses 100 times higher than the recommended level [245]. Additionally, A study investigating the survivability of microencapsulated and non-encapsulated *Salmonella* bacteriophages (BP FGS011) in the gastrointestinal tract found that microencapsulated phages exhibited enhanced resistance to harsh proventriculus-gizzard conditions in vitro compared to free phages. This suggests that microencapsulation may improve phage stability and effectiveness in targeting *Salmonella* within the digestive system [246]. Additionally, A study evaluated a six-phage cocktail for controlling the growth of *Salmonella* strains isolated from swine and poultry, tested in porcine (IPEC-1), avian (CHIC-8E11), and human (HT-29) epithelial cell cultures. Findings indicated that the optimized six-phage formulation demonstrated superior therapeutic potential when used as a prophylactic measure to reduce *Salmonella* colonization across porcine, human, and avian cell lines, outperforming coinfection and remedial treatment strategies [247]. Moreover, an in vitro study demonstrated reductions in *S. enteritidis* counts by 0.65, 1.49, and 0.58 log_10_ CFU/mL following a 3 h co-incubation with each of the three wild-type lytic bacteriophages (LBs) [248]. A significant reduction in the *S. enteritidis* count has also been observed after phage therapy in vivo [248]. Similarly, two *Salmonella* phages, S4lw and D5lw, demonstrated a 3.4–1.7 log_10_ reduction in *S. typhimurium* and *S. enteritidis* following 2–6 h of treatment [249]. Additionally, a survey assessing the sensitivity of 10 *S. enteritidis* and 10 *S. typhimurium* strains to a bacteriophage cocktail demonstrated a significant reduction in *S. enteritidis* and *S. typhimurium* colonization within the crop, liver, spleen, and caeca of broilers at 7, 14, and 21 days following bacteriophage application [250]. Moreover, Anisha et al. (2023) conducted an experimental trial to evaluate the efficacy of a bacteriophage cocktail delivered via feed in reducing *S. typhimurium* colonization in broiler chickens [166]. Birds treated with the phage cocktail exhibited a significant reduction in *S. typhimurium* levels compared to the challenged, untreated group (*Salmonella* was detected in fecal samples from the group receiving 10^5^ PFU/day, while groups receiving 10^6^ and 10^7^ PFU/day showed residual counts of ~4 × 10^2^ CFU/g, compared to ~3 × 10^4^ CFU/g in untreated controls) [251]. Bacteriophages could be combined to make mixtures that can target most common infections. Bacteriophages also could be used alongside antibiotics, showing synergistic effects, especially against infections with antibiotic-resistant bacteria [252]. A significant inhibition of *S. typhi* isolated from stool samples of typhoid patients using a combination of ciprofloxacin and bacteriophages was reported. The Phage–antibiotic synergy was evident on agar plates, with a statistically significant enhancement of bacterial growth inhibition (*p* = 0.03) compared to either treatment [253]. Another study was done to evaluate phage-antibiotic combinations for the treatment of extended-spectrum β-lactamase-producing *S. enteritidis*. A synergistic effect was recorded at 0.25 × MIC of cefixime, gentamicin, ciprofloxacin, and aztreonam antibiotics in combination with phage at 10^4^, 10^6^ and 10^8^ PFU/mL [254].

Despite the promising potential of bacteriophages as targeted therapeutics against MDR pathogens such as *Salmonella*, several challenges persist. Bacterial resistance to phages can emerge through mechanisms like receptor modification and CRISPR-Cas systems, compromising long-term efficacy. Moreover, the narrow host range of many phages limits their utility unless broad-spectrum cocktails are carefully formulated. Regulatory and safety concerns also pose hurdles, as standardized frameworks for phage therapy are still under development, and issues like endotoxin contamination and manufacturing consistency must be addressed. Therapeutic success is further influenced by delivery challenges, including phage survival in hostile environments such as acidic gastric conditions or biofilms, and their pharmacokinetics in vivo. Finally, the scarcity of large-scale clinical trials restricts the broader application of phages in both human and veterinary medicine, despite their demonstrated potential in preclinical studies [255,256,257,258].

### 2.11. Fecal Microbiota Transplantation (FMT)

Fecal microbiota transplantation (FMT) is a process of transferring fecal microorganisms from a healthy host into the intestinal tract of a diseased host [259]. This process can be done through multiple ways, such as colonoscopy, naso-jejunal tube, naso-duodenal tube, or retention enema [67]. FMT is considered a medicinal product in the United Kingdom, a biological product in North America, and a tissue product in different European regions [260]. The intestinal microbiota plays an important role in maintaining a healthy gut and protecting against microbial infections. The FMT approach aims to restore healthy and balanced gut microbiota after unintended disruption by any stressors like pathogenic infection, antibiotic treatment, or dietary change [261]. In November 2022, the FDA has formally approved the first microbiota-based therapy, Rebyota^®^ (Ferring Pharmaceuticals), for preventing *Clostridium difficile* infection recurrence in adults [262,263]. The effect of FMT is multifactorial, involving the host, the microbiota, and the pathogen [264].

Several studies have been investigating the effect of FMT on the *Salmonella* pathogen in vitro and in vivo. For example, Wang et al., investigated the efficacy of FMT from specific pathogen-free (SPF) chickens against *S. enteritidis* infection in chicks. Their findings revealed that FMT successfully inhibited *S. enteritidis* colonization in the liver of challenged chicks. Additionally, FMT significantly increased the relative abundance of Parabacteroides and Bacteroides (*p* < 0.05), key bacterial genera associated with gut health [265]. The treatment also reduced IL-1β and IL-18 levels in serum, suggesting an anti-inflammatory effect. Notably, FMT improved liver inflammatory lesions, strengthened immune response, promoted weight gain, and enhanced overall resistance to SE infection [265]. Another study conducted by Soto et al. found that prolonged ertapenem administration followed by an encapsulated fecal transplant can cure immunocompromised individuals suffering from a GIT infection due to resistant *S. infantis* [264]. FMT is considered a safe approach, with minimal risks and complications; however, there are potential adverse outcomes, including diarrhea, gastrointestinal pain, constipation, and the transmission of pathogenic and antibiotic-resistant microorganisms [266].

### 2.12. Nanomaterials

Nanomaterials or nanoparticles are ultrafine particles that exhibit unique physical and chemical properties, strengthening their use in many biomedical and food quality industries [206]. There are different forms of nanomaterials, such as lipids, polymers, emulsions, proteins, nanobeads, inorganic nanomaterials, etc. [206]. These nanomaterials have been approved and successfully used for controlling infectious and non-infectious diseases. They have the capabilities to inhibit bacterial growth, reduce tissue inflammation, and boost the immune response [206].

Nanomaterials, such as silver nanoparticles (AgNPs), FeO nanoparticles, and chitosan, have shown a significant experimental effect on *Salmonella* infections in animals [267]. An experiment evaluating the antibacterial activity of silver nanoparticles against MDR-*S. enteritidis* showed that AgNPs have reduced the number of viable SE recovered from the challenged SE-infected mouse model [267]. It also noted that the average MIC and MBC of AgNPs were 0.085 ± 0.126 μg/mL and 0.508 ± 0.315 μg/mL, respectively [267]. Moreover, another research has demonstrated that Fe_3_O_4_ magnetic nanoparticles effectively inhibited the viability and invasion of intracellular *S. enteritidis* in chicken hepatocytes [268]. Fe_3_O_4_-NPs were administered orally at 50 mg/kg on days 2, 4, and 6 while *S. enteritidis* was inoculated on day 7. Fe_3_O_4_-NPs increased ROS production and antioxidant enzyme activity, resulting in a significant reduction of the invasion and colonization of *S. enteritidis* in chicken liver [268].

Additionally, an experimental study was done evaluating the effect of Nigella Sativa silver nanoparticles (*NS AgNPs*) on rats infected with *Salmonella* spp. [269]. It was indicated that *NS AgNPs* have reduced the severity of inflammatory responses caused by *Salmonella*. It also did not induce any significant changes in liver and kidney functions. Also, rats infected with *Salmonella* spp. and treated with *NS AgNPs* showed a marked decrease in colony-forming units compared to the untreated infected group [269]. Furthermore, Dolatyabi et al., proposed that orally administering *S. enteritidis* immunogenic outer membrane proteins and flagellin encapsulated in mannose chitosan nanoparticles (OMPs-FLA-mCS NPs) elicited cross-protective mucosal immune responses against *S. typhimurium* colonization in broilers [270]. It also significantly boosted IgA and IgY antibody secretion and resulted in a 0.8 log_10_ CFU reduction in *S. typhimurium* bacterial load within the cecal content [270]. Similarly, A study using Copper/Zinc-modified palygorskite (Cu/Zn-Pal) demonstrated that chickens infected with *S. typhimurium* and treated with Cu/Zn-Pal showed a significant reduction in intestinal colonization [271].

Nanomaterials have also been used as an adjuvant in *Salmonella* vaccines. Incorporating silicon dioxide, iron oxide, carboxymethyl chitosan, and iron oxide-chitosan nanomaterials in the vaccine has improved its efficacy against salmonellosis in chicken, as noted by Ibrahim et al. He found that the use of silicon dioxide SiNPs as a vaccine delivery system could enhance the immune response to *Salmonella* in chickens [272]. That study demonstrated that the use of adjuvanted vaccines with nanomaterials, particularly SiNPs, has significantly increased the protection rate from 67 to 93.3% when compared to the locally used vaccine, which had a protection rate of 83% [272].

Despite nanomaterials’ advantages, concerns remain regarding their long-term safety, potential cytotoxicity, and the possibility of developing bacterial resistance to nanomaterial-based therapies [273], comprehensive in vivo studies and rigorous toxicological assessments are essential to evaluate their biocompatibility, environmental impact, and therapeutic viability before widespread clinical application.

## 3. Potential of Genetic Engineering and CRISPR Technology

Genetic engineering and CRISPR technology hold significant potential for controlling *Salmonella* infections. CRISPR-Cas systems, which are adaptive immune systems found in many prokaryotes, can be engineered to target and edit nucleic acids with high precision [274,275]. This technology can be used to detect and control antibiotic-resistant *Salmonella* strains. It can be used to edit the *Salmonella* genome and potentially disable genes responsible for virulence or antibiotic resistance [276,277]. This approach can help in developing more effective treatments and preventive measures. In addition, CRISPR technology can be employed to combat antimicrobial resistance by re-sensitizing bacterial cells to antibiotics [278]. This can be particularly useful in treating infections caused by multidrug-resistant *Salmonella*. An interesting study was conducted through which the SpvB gene was deleted using CRISPR/Cas9 method from a virulent plasmid of *S. gallinarum* strain (SG18) [279]. The SpvB-deleted *S. gallinarum* strain, when tested for its virulence in broiler chickens, showed no clinical signs or mortality. It was also noted that *S. gallinarum* has lost its ability to invade from the intestine to the liver of broiler chickens [279]. However, despite its promise, the CRISPR-based approach faces several limitations and challenges. Of these challenges is the genetic diversity among circulating *Salmonella* strains, which makes it difficult to design universal CRISPR targets [280]. Editing each strain individually is impractical, especially in field conditions where multiple serovars may coexist. Additionally, efficient delivery of CRISPR components into bacterial populations remains a technical hurdle, particularly in vivo [281]. There are also concerns about off-target effects and the potential for horizontal gene transfer, which could inadvertently spread modified genetic material [282]. Regulatory and ethical considerations further complicate the deployment of gene-editing technologies in food animals. Therefore, while CRISPR offers exciting possibilities, its real-world application for controlling *Salmonella* infections requires careful evaluation and complementary strategies. Researchers are still exploring the use of *Salmonella’s* Endogenous CRISPR-Cas Systems to leverage the bacteria’s natural defense mechanisms to target and eliminate the pathogen [283,284].

## 4. Conclusions and Future Perspectives

*Salmonella* infections continue to pose a significant global health threat due to their zoonotic potential, environmental persistence, and increasing resistance to conventional antibiotics. The widespread misuse of antibiotics in both human and veterinary medicine has not only disrupted host microbiota but also accelerated the emergence of multidrug-resistant *Salmonella* strains, complicating treatment and control efforts. In light of these challenges, there is a growing shift toward alternative antimicrobial strategies that mitigate bacterial virulence without exerting the selective pressure typical of traditional antibiotics.

This review has explored a diverse array of promising alternatives—including probiotics, prebiotics, postbiotics, essential oils, organic acids, vaccines, small molecules, quorum sensing inhibitors, and fecal microbiota transplantation. These agents have demonstrated multifaceted mechanisms of action, such as inhibiting *Salmonella* colonization, biofilm formation, and toxin production, while also enhancing host immune responses. Importantly, several of these alternatives have shown synergistic effects when combined with conventional antibiotics, offering the potential to reduce antibiotic dosages and delay resistance development.

Despite encouraging progress, several challenges hinder the widespread adoption of these alternatives. These include variability in efficacy across *Salmonella* serovars and host species, lack of standardized formulations, safety concerns, and limited clinical validation. Regulatory barriers and gaps in mechanistic understanding further complicate their integration into routine practice.

Future research should prioritize well-controlled in vivo studies, mechanistic elucidation, and translational clinical trials to assess efficacy, safety, and long-term impacts on host health and microbial ecology. Moreover, integrating these strategies into food safety protocols, livestock management systems, and public health frameworks could significantly reduce the burden of MDR *Salmonella*. A multidisciplinary approach—bridging microbiology, pharmacology, veterinary science, and public health—is essential to fully harness the potential of these antimicrobial alternatives and advance toward sustainable, resistance-conscious disease control.

## Figures and Tables

**Table 3:** Therapeutic and prophylactic effect of Probiotics against different *Salmonella* serovars in vitro and in different hosts.

Probiotics	*Salmonella* Serovars	Host	Observations	Refs.
*Bacillus subtilis* and *Bacillus amyloliquefaciens*	*S. hadar, S. enteritidis, S. thompson*	In vitro	*B. subtilis* inhibited 51.1%, 48.3%, and 56.9% of biofilm formation of *S. hadar*, *S. enteritidis*, and *S. thompson*, respectively. Additionally, *B. amyloliquefaciens* inhibited 30.4, 28.6, and 35.5% of biofilm formation of *S. hadar, S. enteritidis,* and *S. thompson*, respectively. No significant reduction in planktonic *Salmonella* cell counts was observed.	[83]
*Lactobacillus animalis* and *Propionibacterium freudenreichii*	*S. typhimurium*	Beef calves	Detection of at least one colony-forming unit (CFU) of *Salmonella* in the feces of commercial beef calves supplemented with *L. animalis* and *P. freudenreichi.*	[84]
* Bacillus. velezensis *	* S. typhimurium *	Mice	* B. velezensis * HBXN2020 reduced *S. typhimurium* in mice feces for approximately 1.3-fold (corresponding to a 0.12 *log*_10_ reduction) to 15-fold (1.18 *log*_10_ reduction) depending on the dosage and timepoint. Additionally, a 1.06, 1.69, 1.14 substantial log reduction in *S. typhimurium* was observed in the cecum, colon, and ileum of mice, respectively, accompanied by notable regulation of cytokine levels (*Tnfa, Il1b, Il6,* and *Il10*). Furthermore, a significant increase in the abundance of beneficial bacteria, including *Lactobacillus* and *Akkermansia*, was recorded, *p* < 0.05.	[85]
*L. fermentum* and *L. acidophilus*	*S. typhimurium*	Mice	Significant upregulating the anti-inflammatory cytokines and downregulating the pro-inflammatory cytokine mRNA induced by *Salmonella* (*p* ≤ 0.05). Also, there was a reduction in *S. typhimurium* counts in mice feces when measured on days 9, 12, and 14 post-infection, *p* < 0.0001.	[86,87]
*L. helveticus* R389	*S. typhimurium*	Mice	Elevated luminal levels of specific anti-*Salmonella* secretory immunoglobulin A (S-IgA), along with a decreased presence of Macrophage Inflammatory Protein-1α (MIP-1α) cells in the lamina propria, were observed. In addition, a reduction in *S. typhimurium* burden in the liver was noted.	[88]
*L. plantarum* and *L. acidophilus*	*S. heidelberg*	Broiler chicken	Substantial reductions in *S. heidelberg* colonization in the ceca by 64% at 24 h, 42% at 96 h, and 46% at 168 h post-treatment.	[89,90]
*L. casei* and *Bifidobacterium breve*	*S. typhimurium*	Broiler chicken	*B. breve* JCM1192 and *L. casei* ATTC334 caused a reduction in *S. typhimurium* colonization in the cecal tonsils by 20% and 10%, respectively.	[91]
*L. rhamnosus* GG	*S. typhimurium*	In vitro	Thermal stress (41 °C for 1 h) significantly increased *S. typhimurium* adhesion to Caco-2 cells by 1.7-fold (*p* = 0.001) and elevated cytotoxicity by 3.5-fold (*p* = 0.0001) as measured by LDH release. Furthermore, pre-treatment with *L. rhamnosus* GG markedly reduced both adhesion (*p* = 0.001) and cytotoxicity (*p* = 0.001) under stress conditions.	[92,93]
*L. rhamnosus* and *B. lactis*	*S. typhimurium*	Mice	An increase in specific anti-*Salmonella* antibodies was observed in both the serum and intestinal tract of mice, indicating a strengthened immune response against the pathogen.	[94,95]
*L. plantarum*, *L. casei*, *L. acidophilus*, and *Enterococcus faecium*	*S. typhimurium*	Horses	In a randomized, double-blind clinical trial involving 130 hospitalized horses, the administration of a probiotic mixture (containing *L. plantarum*, *L. casei*, *L. acidophilus*, and *E. faecium*) reduced the incidence of *Salmonella* fecal shedding after 48 h of hospitalization by approximately 65%. Interestingly, the calculated relative risk (RR) was 0.35, with a 95% confidence interval (CI) of 0.07–1.68, indicating a substantial but statistically cautious reduction in shedding risk.	[96]
*B. thermophilum* RBL67	*S. typhimurium*	In vitro	In co-culture experiments, *S. typhimurium* N-15 exhibited a reduced final cell count of 8.82 ± 0.08 log_10_ CFU/mL, compared to 9.10 ± 0.16 log_10_ CFU/mL in monoculture, indicating that *B. thermophilum* RBL67 modestly suppressed *Salmonella* proliferation. Similarly, RBL67 significantly impacted the transcriptome of *S. typhimurium* through the activation of virulence genes located on *Salmonella* pathogenicity islands 1 and 2 (*SPI*-1 and *SPI*-2).	[97]
*L. acidophilus, L. rhamnosus*, and *Bifidobacterium animalis* subsp. *lactis (Bb12)*	*S. typhimurium*, *S. enteritidis*	Chickens	Supplementing chickens with *L. rhamnosus* via drinking water significantly reduced *S. typhimurium* colonization in the cecum, with bacterial loads decreasing to 5.95 log CFU/g at 7 days post-infection (dpi) and 3.74 log CFU/g at 14 dpi compared to higher levels in the untreated control group. Furthermore, intestinal morphology improved notably, with increased villus height and a higher villus height-to-crypt depth ratio observed in LGG-treated birds, indicating enhanced nutrient absorption and gut integrity. In addition, LGG supplementation also modulated gut microbiota, enriching beneficial genera such as *Butyricicoccus*, *Erysipelatoclostridium*, *Flavonifractor*, and Bacillus. Interestingly, immune responses were positively influenced, showing reduced inflammatory markers and upregulated mucosal immunity.	[98]
*E. coli Nissle* 1917	*S. pullorum*	Egg laying chickens	The study examined cellular invasion of *S. pullorum* in a chicken fibroblast cell model and found that pretreatment with EcN significantly decreased cellular invasion.	[99]
*E. faecium* Smr18	*S. typhi*	Mice	*E. faecium* Smr18 administration significantly increased serum levels of nitrate by 1.63-fold and 3.22-fold in the pre- and post-administration groups, respectively. This increased level of nitric oxide intermediates facilitated the clearance of *S. typhi* from the host.	[100]
*Pediococcus pentosaceus*	*S. enteritidis*	Broiler chicken	*P. pentosaceus* significantly reduced the mortality rate of chickens infected with *S. enteritidis* by 23.3% compared to 44.4% of the control group (infected but no probiotic). Moreover, the *Salmonella* count in the caecum of the control group rose quickly, peaking at 3 days post-infection (dpi), while in the experimental group (with *P. pentosaceus*), the increase was more gradual, and overall, *Salmonella* counts were significantly lower than those of the control group.	[101]
*L. reuteri* KUB-AC5	*S. typhimurium*	Mice	*L. reuteri* KUB-AC5 significantly reduced the severity of gut inflammation induced by *S. typhimurium* compared to that of untreated mice. Likewise, AC5 significantly reduced the gene expression of pro-inflammatory cytokines such as Kc, Il-6, and IFN-γ in the mouse colon, cecum, and ileum. Also, the tight junction protein gene Zo-1 expression was upregulated in the gut of AC5-fed mice.	[102]
*P. pentosaceus*	*S. typhimurium*	Mice	*P. pentosaceus* modulates the cell-mediated immune responses by up-regulation of the gene expression of the proinflammatory cytokines IFN-γ and TNF-α” in the small intestine.	[103]
*E. faecium NCIMB* 11181	*S. typhimurium*	Broiler chicken	A significant inhibition of *Salmonella* intestinal colonization and translocation was observed (*p* < 0.05), along with a reduction in intestinal cell apoptosis and gut damage caused by *S. typhimurium* infection. Similarly, a notable increase in anti-*Salmonella* antibodies was detected in both the serum of infected birds and the intestinal mucosa (*p* < 0.05).	[104]
*B. subtilis* and *B. coagulans*	*S. typhimurium*	Rats	A significant reduction in *Salmonella* infiltration into the lymph nodes, liver, and spleen was observed, accompanied by decreased oxidative stress, inflammatory mediators, and alterations in biochemical and hematological parameters.	[105]
*B. pumilus* SMU5927	*S. enteritidis*	Mice	*B. pumilus* enhanced intestinal morphology and strengthened the intestinal barrier function, contributing to improved gut integrity. Additionally, a significant increase (*p* < 0.05) in the alpha diversity of beneficial intestinal microbiota was observed.	[106]
*B. subtilis* LF11	*S.* braenderup	Broiler chicken	A decrease in *S. braenderup* adhesion and invasion to the NCM460 cells was observed, leading to an increase in NCM460 cell survival. In addition, a significant reduction in IL-8 production was noted, along with the downregulation of gene transcription for proinflammatory cytokines IL-6, IL-8, and TNF-α.	[107]
*Pediococcus acidilactici*	*S. typhimurium*	In vitro	Both bacteriocin K10 and bacteriocin HW01 significantly inhibited *S. typhimurium* biofilm formation.	[108]
*E. faecium* 669	*S. enteritidis*	Broiler chicken	Enhanced gut integrity with significant reduction in *S. enteritidis* colonization and shedding in treated broiler chickens’ cecum.	[109]
*E. faecium*	*S. typhimurium*	weaning piglets	A significant elevation in serum IgM and IgA levels targeting *S. typhimurium* was observed.	[110]
*L. reuteri* and *E. faecium*	Multidrug-resistant *S. typhimurium* and *S. enteritidis*	In vitro	A decrease in mucin adhesion and biofilm formation was observed in cephalosporin- and fluoroquinolone-resistant *S. typhimurium* and *S. enteritidis*.	[111]
*B. infantis* and *L. Rhamnosus* combined with oligofructose-enriched inulin	*S. typhimurium*	Weaned Piglets	Both probiotic strains and the prebiotic mixture offered benefits in mitigating *S. typhimurium* infection in weaned piglets. Along with that, probiotic combination appears to enhance pathogen clearance, while the prebiotic mixture may reduce colonic colonization and modulate the immune response.	[112]
*P. pentosaceus* GT001	*S. typhimurium*	Broiler chicken	*P. pentosaceus* GT001 significantly improved growth performance, immune function, antioxidant status, and microbial balance in broiler chickens challenged with *S. typhimurium*. Additionally, probiotic-treated birds showed greater body weight gain (*p* < 0.05), elevated serum levels of T-AOC, SOD, CAT, and GSH-Px, and reduced MDA and liver enzymes compared to infected controls. Immunoglobulin levels (IgA, IgG, IgM) and cytokines (IL-6, IL-10) were also enhanced (*p* < 0.05), while *Salmonella* load in the ceca dropped below detectable levels within 14 days.	[113]

**Table 4:** Influence of various forms of prebiotics on different *Salmonella* serovars in different hosts.

Prebiotics	*Salmonella* Serovars	Host	Observations	Ref.
FOS combined with Alfalfa	*S. enteritidis*	Laying Hens	Significant reduction in *S. enteritidis* colonization in ovaries and livers (*p* ≤ 0.05). Significant decreases in cecal *S. enteritidis* counts were also observed.	[126]
Non-starch soluble polysaccharide from plantain (Plantain NSP)	*S. typhimurium*	In vitro	Plantain NSP significantly reduced *S. typhimurium* adhesion to Caco2 cells (85.0 ± 8.2%, *p* < 0.01) and inhibited its invasion into these cells (80.2 ± 9.7%). Additionally, it effectively blocked the translocation of *S. typhimurium* across M-cells and Peyer’s patches, suggesting a protective role in intestinal barrier integrity.	[127]
β-galactomannan (βGM), MOS	*S. typhimurium*	In vitro	Both βGM and Mannan-oligosaccharide decreased the secretion of IL6 and CXCL8 induced by *Salmonella,* with inhibition of *Salmonella* adhesion to intestinal epithelial cells.	[128]
FOS	*S. typhimurium*	Pigs	A decrease in the shedding of *S. typhimurium* was observed, indicating a potential reduction in pathogen transmission and environmental contamination.	[129]
β-galactomannan oligosaccharide (β-GMOS)	*S. typhimurium*	Fattening pigs	Supplementing pig feed with at least 2 kg t^−1^ of β-GMOS during the fattening period was linked to a decrease in *S. typhimurium* prevalence, shedding, and seroconversion.	[130]
Bio Mos^TM^	*S. enteritidis*	Broiler chickens	No substantial impact was observed on the performance of broiler chickens challenged with *S. enteritidis* and also no significant effect on the production of anti-*S. enteritidis* antibodies.	[131]
β-glucan	*S. typhimurium* var. Copenhagen	Weaning piglets	β-glucan did not effectively prevent *S. typhimurium* colonization; however, it may contribute to reducing pathogen transmission among pigs (it significantly lowers cecal pathogen load and reduces fecal shedding, which helps minimize transmission among pigs).	[132]
FOS & inulin	*S. enteritidis*	chicken macrophages	A significantly reduced number of viable intracellular *S. enteritidis* was observed in HD11 cells, accompanied by a substantial decrease in IL-1β expression, indicating a potential modulation of inflammatory response.	[133]
FOS, XOS & apple pectin	*S. typhimurium*	Mice	Supplementation with 10% FOS or XOS resulted in increased translocation of *S. typhimurium* SL1344 to internal organs in mice, whereas the inclusion of 10% apple pectin led to a higher *S. typhimurium* count in both intestinal content and feces.	[125]

**Table 6:** Commercially available vaccines against Salmonellosis.

Vaccines	Targeted *Salmonella* Serovars	Host	Observations	Refs.
EnterVene-d; Boehringer Ingelheim Vetmedica Inc., Duluth, GA, USA	* S. dublin *	Dairy calves	Vaccinated cows had significantly higher *S. dublin* antibody titers at calving (40.3 ± 9.1) compared to controls, and their calves showed elevated antibody levels (88.5 ± 8.9) after receiving colostrum, compared to calves from unvaccinated cows.	[225]
*Salmonella* Newport extract vaccine, Zoetis Inc., Kalamazoo, MI, USA	*S. newport*, *S. montevideo*, and *S. anatum*	Cattle	A significant decrease in *Salmonella* prevalence was detected in the sub-iliac and pre-scapular lymph nodes (*p* = 0.05), as well as across all lymph nodes combined (*p* = 0.04).	[226]
Modified-live *S. typhimurium* vaccine (PoulvacR ST; Zoetis Inc., Madison, NJ, USA)	*S. enteritidis*, *S. kentucky*, *S. typhimurium*, *S. heidelberg*, and *S. hadar*	Chicken	A 50% reduction in *S. enteritidis*, *S. kentucky*, *S. typhimurium*, *S. heidelberg*, and *S. hadar* were observed in the liver and spleen of chickens.	[227]
Nobilis^®^ Salenvac ETC	*S. enteritidis*, *S. typhimurium*, *S. infantis*, and *S. hadar*	Chicken	Significant reductions in fecal shedding were observed for *S. enteritidis* (*p* = 0.001), *S. typhimurium* (*p* = 0.0055), *S. infantis* (*p* = 0.0299), and *S. hadar* (*p* = 0.0013). Likewise, notable decreases in organ invasion were reported for *S. enteritidis* (*p* = 0.001), *S. typhimurium* (*p* = 0.001), and *S. infantis* (*p* = 0.0014), whereas the reduction for *S. hadar* was not statistically significant (*p* = 0.347).	[228]
AviPro^®^ *Salmonella* Vac E, Elanco, Greenfield, IN, USA	*S. enteritidis*	Chicken	Cloacal shedding of *S. enteritidis* was reduced to 8.3% compared to 66.7% in the unvaccinated control group during the 29-week challenge. Organ colonization (liver, spleen, ovaries) was also significantly lower in vaccinated hens, with 0–8.3% positive samples versus 58.3–75% in controls. Also, eggshell contamination dropped to 0% in vaccinated birds, while the control group had contamination rates of 16.7% and 25% in trials 4 and 5, respectively.	[229]
Chitosan-adjuvanted *Salmonella* subunit nanoparticle vaccine (OMPs-F-CS NPs)	*S. enteritidis*	Chicken	Serum IgG levels were approximately 2.5-fold higher in vaccinated groups. There was also a marked upregulation of immune-related cytokines such as IFN-γ, IL-17, and TGF-β in the cecal tonsils of vaccinated birds. Vaccinated birds exhibited a 1.5 to 2 log_10_ reduction in bacterial load in internal organs (like liver and spleen) and feces compared to controls.	[230]
Endovac-Bovi^®^ with Immune Plus^®^ (IMMVAC Inc., Columbia, MO, USA)	*S. newport*, *S. montevideo*, and *S. anatum*	Cattle	A significant reduction in *Salmonella* prevalence was observed in the right popliteal lymph node (*p* = 0.03). Additionally, a decrease in *Salmonella* prevalence was noted across both popliteal lymph nodes (*p* = 0.09).	[226]
Autologous killed tri vaccine	*S. typhimurium*, *S. agona*, *S. mbandaka*, *S. infantis*, *S. orion*, and *S. zanzibar*	Chicken	The vaccine induced a highly significant antibody response (*p* < 0.001), with 39% of maternal antibodies detected in 16% of egg yolks, indicating the possibility of passive immunity transfer to offspring.	[231]
Vivotif^®^ (Typhoid Vaccine Live Oral Ty21a)	*S. paratyphi* A, B	Human	The vaccine showed an efficacy of 51% in adults and children over five years old, offering meaningful cross-protection against both *S. typhi* and *S. paratyphi*.	[232,233]
ViCRM_197_ conjugate vaccine, Novartis, Basel, Switzerland	*S. typhi*	Human	A marked rise in IgG antibody titers was recorded, correlating with an 89% protective efficacy against typhoid fever, with immunity sustained for a minimum duration of four years.	[234]
Vi Conjugate (Vi-rEPA)	*S. typhi*	Human	A rapid secretion of *Vi*-specific IgM and IgG antibodies was observed, accompanied by a two-log reduction in *S. typhi* shedding.	[235]

## Data Availability

All data was included in the published paper.
